# Premature mortality in Belgium in 1993-2009: leading causes, regional disparities and 15 years change

**DOI:** 10.1186/2049-3258-72-34

**Published:** 2014-10-01

**Authors:** Françoise Renard, Jean Tafforeau, Patrick Deboosere

**Affiliations:** Department Public Health and Statistics, Institute of Public Health, Brussels, Belgium; Interface Demography, Section Social Research, Free University of Brussels, Brussels, Belgium

**Keywords:** Premature mortality, Mortality rates, Potential Years of Life Lost, Causes of death, Belgium

## Abstract

**Background:**

Reducing premature mortality is a crucial public health objective. After a long gap in the publication of Belgian mortality statistics, this paper presents the leading causes and the regional disparities in premature mortality in 2008–2009 and the changes since 1993.

**Methods:**

All deaths occurring in the periods 1993–1999 and 2003–2009, in people aged 1–74 residing in Belgium were included.

The cause of death and population data for Belgium were provided by Statistics Belgium , while data for international comparisons were extracted from the WHO mortality database.

Age-adjusted mortality rates and Person Year of Life Lost (PYLL) were calculated. The Rate Ratios were computed for regional and international comparisons, using the region or country with the lowest rate as reference; statistical significance was tested assuming a Poisson distribution of the number of deaths.

**Results:**

The burden of premature mortality is much higher in men than in women (respectively 42% and 24% of the total number of deaths). The 2008–9 burden of premature mortality in Belgium reaches 6410 and 3440 PYLL per 100,000, respectively in males and females, ranking 4th and 3rd worst within the EU15. The disparities between Belgian regions are substantial: for overall premature mortality, respective excess of 40% and 20% among males, 30% and 20% among females are observed in Wallonia and Brussels as compared to Flanders. Also in cause specific mortality, Wallonia experiences a clear disadvantage compared to Flanders. Brussels shows an intermediate level for natural causes, but ranks differently for external causes, with less road accidents and suicide and more non-transport accidents than in the other regions.

Age-adjusted premature mortality rates decreased by 29% among men and by 22% among women over a period of 15 years. Among men, circulatory diseases death rates decreased the fastest (-43.4%), followed by the neoplasms (-26.6%), the other natural causes (-21.0%) and the external causes (-20.8%). The larger decrease in single cause is observed for stomach cancer (-48.4%), road accident (-44%), genital organs (-40.4%) and lung (-34.6%) cancers. On the opposite, liver cancer death rate increased by 16%.

Among female, the most remarkable feature is the 50.2% increase in the lung cancer death rate. For most other causes, the decline is slightly weaker than in men.

**Conclusion:**

Despite a steady decrease over time, international comparisons of the premature mortality burden highlight the room for improvement in Belgium. The disadvantage in Wallonia and to some extent in Brussels suggest the role of socio-economic factors; well- designed health policies could contribute to reduce the regional disparities. The increase in female lung cancer mortality is worrying.

**Electronic supplementary material:**

The online version of this article (doi:10.1186/2049-3258-72-34) contains supplementary material, which is available to authorized users.

## Background

Reducing premature deaths is a crucial goal of public health policies [1]; premature mortality statistics are invaluable indicators to assist important public health functions: the establishment of priorities, the monitoring of temporal trends to evaluate the impact of public health actions and the detection of geographical disparities that can highlight problems in resources allocations or non-medical health determinants [2, 3].

Traditional age-adjusted mortality rates allow for temporal or geographical comparisons of premature death figures. However, as stated by the US Centre for Diseases Control and Prevention (CDC) [2], death rates often fail to tell the entire story of premature mortality: since most deaths occur in older age-groups, mortality rates are dominated by the underlying diseases of the elderly. An alternative measure has been proposed [2, 4, 5], the “Potential Years of Life Lost (PYLL)”, an indicator weighting the deaths occurring at younger ages more heavily than those occurring later. It has been largely used as a planning tool since the 1980s.

In Belgium, previous studies analysed the cause-specific (premature) mortality either using rates or PYLL [6–9]. The latest published studies refer to years up to 1997; afterwards, there has been a long hiatus in the publication of mortality data due to a reorganisation of the responsibilities in processing the death certificates. For more than 12 years, Belgian mortality data were only partially available on the regional levels. Recently, causes of deaths data have been produced again at the national level. Those are currently (December 2013 ) available for nearly all years previous to 2009 (except for 2000–2002). This prompted us to analyse the recent patterns of premature mortality. The current paper presents the 2008–9 premature mortality figures using both death rates and PYLL; the aim is to identify the leading causes of premature death, to look at regional disparities and to monitor the changes since 1993. In addition the overall Belgian premature mortality level is compared to the EU15 countries.

## Methods

### Period and people under study

Premature mortality is defined here as death occurring before 75 years. All deaths occurring in the periods 1993–1999 and 2003–2009 (last available years at national level in December 2013), in people aged 1 to 74 years and residing in Belgium were included. Infant deaths are excluded from the study.

**Cause of death and population data for Belgium** are provided by Statistics Belgium, Directorate in charge of the production of vital statistics at the national level. The causes of death are specified in deaths certificates by a medical doctor; they are subsequently coded according to the ICD rules by trained staff within 2 regional Health Agencies (one for Flanders and Brussels, one for Wallonia), before being pooled at the national level by Statistics Belgium. Regular coordination meetings between the regional and federal levels are organised in order to guarantee consistency in the coding/registration rules.

**Deaths and population data for International comparisons:** deaths and population data by sex and 5 years age groups for the EU15 countries were extracted from the WHO Mortality Database to compare overall PYLL rates.

**Classifications in use**: the 9th revision of the International Classification of Disease (ICD-9) was in use until 1997 while the ICD-10 was introduced in 1998 [10, 11]. Causes of deaths were poorly coded (except for neoplasms) in the first 2 years after the adoption of the 10th revision (1998–1999); consequently for those 2 years only overall and cancer mortality will be published. The transition in ICD-classification created also jumps in the subclasses of circulatory diseases; hence those will not be used in the trends analysis. As suicide is often suspected being under-reported, several correction methods have been proposed [12–15]. We will, in addition to the registered suicide data, calculate estimations by using Jougla’s method [13].

**Grouping of the causes of death:** the causes of death are displayed according to the ICD-chapters. Chapters with less than 200 yearly cases for Belgium are not shown separately. Besides, single causes or group of causes are presented for cancers, circulatory diseases and external causes, as well as for some single causes also relevant for health promotion (chronic liver diseases, COPD). The classification used along with ICD-9 and ICD-10 is summarized in Table 1.

**Table 1:** **ICD-9 and ICD10 codes for the categories and single causes of deaths used**

	ICD chapter	Selected single causes or group of causes	ICD9	ICD10	Clusters of main causes (used in Table2)
**Natural causes**	Inf.&parasit.diseases		1-139	A00-B99	
	Neoplasms		140-239	C00-D48	Neoplasms
		Head,neck& oesoph.Ca	140-149;161;150	C00-C14;C30-C32;C15	
		Stomach Ca	151	C16	
		Colorectal Ca	153;154.0;154.1	C18-C20	
		Liver Ca	155	C22	
		Pancreas Ca	157	C25	
		Lung Ca	162	C33-C34	
		Female Breast Ca	174	C50	
		Female genital org.Ca	179-184	C51-C58	
		Male genital org.Ca	185-187	C60-C63	
		CNS Ca	191-192	C70-C72	
		Hematol.Ca	200-208	C81-C96	
		Other Neoplasms			
	Endocrine dis		240-279	E00-E99	
	Mental&Neurol Dis.		290-319; 320-389	F00-F99; G00-G99	
	Circulatory diseases		390-459	I00-I99	Circulatory diseases
		Isc.Heart Dis.			
		Cerebrov.dis.&HTA			
		Other Circ.Dis.			
	Resp.Syst.dis.		460-519	J00-J99	All Other natural causes
		Chron.Obs.Pulm.Dis.			
	Digest.Syst.Dis.		520-579	K00-K93	
		Chronic Liver Dis.			
	Genito-urin.Syst.Dis		580-629	N00-N99	
	Sympt.&Ill-def.		780-799	R00-R99	
**External causes**	External causes		E800-899	V01-Y98	External causes
		Road Accident	E810-829	V01-V80;V82-V89,V99	
		Non transp.acc.(Poison/fall/envir.)	E850-859;E880-888; E890-929	X00-X49;W00-W99;	
		Suicide	E950-959	X60-X84	
		Event of Undet.Intent Miscell. other external causes**	E980-989 Remaining ext. codes	Y10-Y34 Remaining .ext. codes	

*Chapters with less than 200 yearly cases were not displayed.

**Miscellaneous other external causes include non-road transport accidents (train, plane, water), homicide, iatrogenic, legal interventions and war.

**Indicators:** age-standardised death rates (1–74 year) are computed with the direct method [16]. Potential Years of Life Lost (PYLL), at population level, between 1 and 74 years are computed as the summation of the remaining years, had the deceased lived up to 75 years. Individual age at death was used for the computation. The age adjusted PYLL rates per 100.000 are calculated according to the method described by Romeder [5]. Mortality rates and PYLL-rates used for Belgian regional or time-trends comparisons are age-adjusted with the 2000 Belgian population as a standard, while for EU15 comparisons, the European Standard population [17] is used.

### Comparisons and statistical analysis

$$\mathrm{Var}\;\left(\mathrm{ASR}\right)\;=\;\sum \left(a_{i}{w_{i}}^{2}*\;100.000/n_{i}\right)\;/\;{\left(\sum w_{i}\right)}^{2}$$

- Cause specific mortality rates were compared between the regions and between EU15 countries with the Rate Ratios of the age-adjusted mortality rates, using the region/country with the lowest rate as reference (Flanders, Sweden Spain). To test for the statistical significance, we first calculated the variance of the age-adjusted rates using a Poisson approximation as [16, 18]:

A z-test [19] was then performed as *z = (ASR1-ASR2)/SQRT (Var (ASR1) + Var(ASR2))*

*The 95*% *confidence interval of the age-adjusted rates was also computed for the international rates.*

Where ASR = Age Standardized rates; a_i_ = age- specific rates per 100.000 in the age-group i; n_i_ = effectif of the population for the age group i; w_i_ = effectif of the standard population for the age group i, var(ASR) = variance of the age-standardized rates.

Beside the statistical comparison, the EU countries were ranked by sex based on the burden of overall premature mortality expressed in PYLL; the causes of deaths were also ranked by region in function of their burden expressed in PYLL.

The evolution between the 1st and 4th period is expressed as a relative rate difference (Rate 4th –Rate 1st)/rate 1st; the statistical significance was tested with a z-test calculated the same way as for regional comparisons.

**All analyses** were performed using SAS 9.3 statistical software.

## Results

### Overall premature mortality and share of the main categories of causes of death in 2008–9

Among the 207.571 deaths that occurred in Belgium in 2008–2009, we focused here on the 69.571 deaths (33.5% of the total) that were non-infant premature deaths (1–74 yr); the remaining deaths were infant deaths (0.4% of all deaths), and deaths occurring in people aged 75 and over (66.1%). While the total number of deaths is similar in both sexes, the fraction of those deaths that occurs prematurely (as defined here before 75) is much higher in men than in women, representing 42% and 24% of the total deaths respectively.

Belgium ranks 4th worst for premature mortality in males and 3rd worst for females in the EU15 (ranked on PYLL) (Table 2) with a quite similar ranking based on rates. Compared to the countries with the lowest rates (Sweden and Spain, respectively for men and women) the premature mortality Rate Ratios for Belgium are 1.31 in men and 1.40 in women.

**Table 2:** **Premature mortality (1–75 yr) in EU-15 countries, 2008–2009 Person-years of Life Lost, Rates and Ranking**

*Country*	*PYLL 1-75*	*RANK Pyll 1-75*	*RATES 1-75*	*RANK Rates 1-75*	*RATIO Rates 1-75*	*95% CI Rates 1-75*
***Sex = Males***						
Finland	7177	1	437.6	1	1.45	[432.1; 443.0]
Portugal	6997	2	428.9	3	1.42	[425.0; 432.8]
France	6477	3	392.1	5	1.30	[390.5; 393.7]
Belgium	6211	4	396.5	4	1.31	[392.7; 400.2]
Greece	6183	5	379.6	7	1.26	[376.1; 383.1]
Denmark	6151	6	429.5	2	1.42	[424.1; 434.9]
Austria	5817	7	378.9	8	1.25	[374.8; 383.0]
Ireland	5798	8	372.1	9	1.23	[366.0; 378.3]
Germany	5751	9	388.3	6	1.29	[387.0; 389.6]
United Kingdom	5628	10	362.6	10	1.20	[361.1; 364.1]
Spain	5476	11	358.9	11	1.19	[357.2; 360.7]
Luxembourg	4862	12	330.5	12	1.09	[314.1; 347.0]
Italy	4727	13	314.2	14	1.04	[312.9; 315.6]
Netherlands	4544	14	326.0	13	1.08	[323.3; 328.7]
Sweden	4518	15	302.1	15	1.00	[298.7; 305.5]
EU15 mean	5661	.	367.8	.	.	[367.2; 368.4]
***Sex = Females***						
***Country***	***PYLL 1-75***	***RANK Pyll 1-75***	***RATES 1-75***	***RANK Rates 1-75***	***RATIO Rates 1-75***	***95% CI Rates 1-75***
Denmark	3724	1	272.0	1	1.78	[267.8; 276.2]
United Kingdom	3403	2	227.8	2	1.50	[226.7; 229.0]
Belgium	3341	3	213.9	4	1.40	[211.2; 216.5]
Ireland	3259	4	216.5	3	1.42	[211.9; 221.2]
Netherlands	3137	5	211.0	5	1.38	[208.8; 213.1]
Finland	3086	6	194.3	7	1.27	[190.8; 197.8]
Portugal	3063	7	192.9	8	1.27	[190.4; 195.3]
Germany	3008	8	201.5	6	1.32	[200.6; 202.4]
France	2953	9	177.3	12	1.16	[176.3; 178.4]
Austria	2861	10	188.9	10	1.24	[186.1; 191.7]
Sweden	2736	11	189.8	9	1.25	[187.1; 192.4]
Greece	2544	12	164.5	13	1.08	[162.3; 166.7]
Luxembourg	2514	13	182.5	11	1.20	[170.5; 194.5]
Italy	2425	14	162.8	14	1.07	[161.9; 163.7]
Spain	2422	15	152.4	15	1.00	[151.3; 153.5]
EU15 mean	2912	.	190.2	.	.	[189.8; 190.6]

*Rates and Pyll are expressed per 100.000 Person-Years and age-standardized on European population* *Author's own calculation.*

Table 3 displays the age-adjusted death rates (1–74 years), PYLL and share by sex for the main categories of conditions (neoplasms, circulatory diseases, all other natural causes, and external causes) in 2008–9. Male to female ratio is nearly 2 for the overall mortality rates/PYLL, but varies with the specific causes, ranging from 1.6 and 1.3 (as expressed respectively in rates or in PYLL) for neoplasms to 2.6 and 3.0 for external causes.

**Table 3:** **Major groups of premature death (1–74 yr), Belgium 2008–2009 Age-adjusted rates and Potential Years of Life Lost**

	*Males*				*Females*				*Sex ratio*	
	*Age-adj. RATES*	*%*	*Age-adj.PYLL*	*%*	*Age-adj. RATES*	*%*	*Age-adj.PYLL*	*%*	*RATES*	*PYLL*
*NEOPLASMS*	175.2	37.6	1914.1	29.9	111	44.2	1444.9	42.0	1.6	1.3
OTH. NAT. CAUSES*	120	25.7	1524.9	23.8	67.5	26.9	879.2	25.6	1.8	1.7
*CIRCUL. DISEASES*	109.6	23.5	1176.2	18.4	49	19.5	509.6	14.8	2.2	2.3
*EXTERNAL CAUSES*	61.5	13.2	1795.3	28.0	23.8	9.5	605.9	17.6	2.6	3.0
*TOTAL*	466.3	100.0	6410.4	100.0	251.2	100.0	3439.6	100.0	1.9	1.9

*Oth. Nat. causes = all natural causes except Neoplasms and Circulatory Diseases.

Neoplasms represent the first group of premature mortality, both in men and women. The next cause depends on the indicator selected: using mortality rates, the mixed group of ”all other natural causes” ranks second, followed by circulatory diseases and external causes, the latter representing 13.2% and 9.5% in males and females respectively. However, when using PYLL, external causes represent respectively 28.0% and 17.7% in males and females. This shift is due to the fact that most external causes of death occur in younger people.

### Regional differences in overall and cause-specific premature deaths

Important regional differences are observed both in overall and in cause-specific premature mortality (Tables 4 and 5). Flanders has the lowest overall premature deaths rates in both sexes (409.6 per 100.000 in males and 224.4 per 100.000 in females). Overall rates in Brussels and Wallonia are respectively 20 and 40% higher in men and 20 and 30% higher in women compared to Flanders.

**Table 4:** **Regional Comparisons of Causes of Premature deaths in Males, Belgium 2008–2009**

*Causes of death grouped*	*Detailed cause*	*RATES Belgium*	*PYLL Belgium*	*RATES Flanders*	*PYLL Flanders*	*RATES Brussels*	*PYLL Brussels*	*Rates ratio Bxl/Fla*	*RATES Wallonia*	*PYLL Wallonia*	*Rates ratio Wal/Fla*
ALL PREMATURE		466.3	6410	409.6	5491	492.9	6521	1.2**	572.2	8161	1.4**
NATURAL CAUSES		404.7	4615	356.0	3883	436.8	5070	1.2**	493.2	5894	1.4**
ALL NEOPLASMS		175.1	1914	164.0	1735	176.4	2069	1.1*	196.4	2210	1.2**
	*Head, Neck & oesoph.Ca*	16.6	219	15.6	199	17.0	220	1.1	18.6	258	1.2**
	*Stomach Ca*	5.1	56	4.9	52	6.6	95	1.3	5.0	53	1.0
	*Colorectal Ca*	15.6	152	15.2	143	15.8	169	1.0	16.3	163	1.1
	*Liver Ca*	5.9	64	4.8	53	7.7	89	1.6*	7.6	80	1.6**
	*Pancreas Ca*	9.0	92	8.3	83	10.3	105	1.2	10.0	107	1.2*
	*Lung Ca*	61.9	630	58.2	564	56.8	625	1.0	70.1	754	1.2**
	*Male genital org.Ca*	8.8	65	8.4	61	8.5	64	1.0	9.6	73	1.1
	*CNS Ca*	5.9	106	6.3	111	4.9	86	0.8	5.4	104	0.9
	*Hematol.Ca*	11.5	138	11.3	135	10.7	155	1.0	12.2	141	1.1
	*Other Neoplasms*	34.9	391	31.0	336	38.0	462	1.2*	41.6	477	1.3**
CIRCULATORY DISEASES		109.6	1176	99.3	1032	115.6	1257	1.2**	129.0	1434	1.3**
	*Isc.Heart Dis.*	47.6	512	42.1	431	53.6	571	1.3**	57.0	656	1.4**
	*Cerebrov.dis.&HTA*	20.7	209	18.4	185	21.5	239	1.2	25.3	249	1.4**
	*Other Circ.Dis.*	41.3	455	38.8	416	40.4	447	1.0	46.7	529	1.2**
ALL OTH.NAT.CAUSES		120.0	1525	92.8	1116	144.8	1743	1.6**	167.9	2250	1.8**
Inf.&parasit.diseases		8.6	116	6.1	79	13.1	187	2.1**	12.6	166	2.1**
Endocrine dis		9.1	122	6.6	98	13.2	163	2.0**	13.3	160	2.0**
Mental&Neurol Dis.		22.7	345	18.3	249	21.8	276	1.2	31.7	546	1.7**
Resp.Syst.dis.		36.3	315	29.1	237	43.8	370	1.5**	48.9	451	1.7**
	*Chron.Obs.Pulm.Dis.*	21.5	170	16.8	122	25.7	200	1.5**	30.0	254	1.8**
Digest.Syst.Dis.		24.8	356	19.1	250	28.5	436	1.5**	34.9	534	1.8**
	*Chronic Liver Dis.*	13.5	217	9.8	145	14.8	253	1.5**	20.2	342	2.1**
Genito-urin.Syst.Dis		4.0	34	2.8	23	5.6	41	2.0*	5.9	55	2.1**
Sympt.&Ill-def.		10.4	158	7.4	104	13.8	189	1.9**	15.5	252	2.1**
EXTERNAL CAUSES		61.5	1795	53.6	1608	56.2	1451	1.0	79.0	2267	1.5**
	*Road Accident*	14.4	527	12.8	469	5.8	210	0.5**	20.1	739	1.6**
	*Non transp.acc.(Poison/fall/envir.)*	15.8	358	12.8	304	18.6	403	1.5**	20.8	450	1.6**
	*Suicide*	26.2	760	23.8	717	18.5	483	0.8**	33.3	937	1.4**
	*Event of Undet.Intent*	2.9	85	2.5	71	9.3	254	3.7**	1.9	54	0.8
	*Miscell.oth.Ext.causes*	2.3	65	1.7	47	4.0	101	2.3**	2.9	88	1.7**

*Age-adjusted rates and Potential Years of Life Lost rates per 100.000.*

*ICD Chapters with fewer than 200 cases a year are not displayed.

Significance levels of the z test: '**' = p <0.01; '*' = p <0.05; 'ns' = Not significant.

**Table 5:** **Regional Comparisons of Causes of Premature deaths in Females, Belgium 2008–2009**

*Causes of death Grouped*	*Detailed Cause*	*RATES Belgium*	*PYLL Belgium*	*RATES Flanders*	*PYLL Flanders*	*RATES Brussels*	*PYLL Brussels*	*Rates ratio Bxl/Fla*	*RATES Wallonia*	*PYLL Wallonia*	*Rates ratio Wal/Fla*
ALL PREMATURE		251.2	3440	224.4	3078	275.8	3757	1.2**	295.3	4019	1.3**
NATURAL CAUSES		227.4	2834	203.2	2526	250.5	3137	1.2**	267.0	3317	1.3**
ALL NEOPLASMS		111.0	1445	106.1	1398	120.0	1548	1.1**	118.1	1506	1.1**
	*Head, Neck & oesoph.Ca*	3.6	46	3.1	41	4.5	60	1.4	4.3	52	1.4*
	*Stomach Ca*	2.2	30	2.0	30	2.3	38	1.1	2.4	27	1.2
	*Colorectal Ca*	9.8	107	9.7	104	10.8	126	1.1	9.8	105	1.0
	*Liver Ca*	2.4	23	2.1	20	3.3	37	1.6	2.7	25	1.2
	*Pancreas Ca*	5.5	57	5.3	59	6.8	70	1.3	5.7	50	1.1
	*Lung Ca*	19.7	248	17.0	211	23.3	287	1.4**	23.9	306	1.4**
	*Breast Ca*	25.2	364	25.6	369	26.7	407	1.0	24.2	345	0.9
	*Female genital org.ca*	13.4	171	13.2	174	14.3	185	1.1	13.5	162	1.0
	*CNS Ca*	4.0	76	4.3	81	3.0	45	0.7*	3.7	76	0.9
	*Hematol.Ca*	7.4	99	7.4	107	6.6	83	0.9	7.7	91	1.0
	*Other Neoplasms*	17.7	223	16.4	202	18.4	209	1.1	20.2	266	1.2**
CIRCULATORY DISEASES		49.0	510	43.8	436	53.1	560	1.2**	57.5	626	1.3**
	*Isc.Heart Dis.*	15.1	142	13.8	124	16.7	154	1.2	17.0	171	1.2**
	*Cerebrov.dis.&HTA*	13.9	150	12.6	132	15.2	158	1.2	15.9	180	1.3**
	*Other Circ.Dis.*	20.0	218	17.4	180	21.1	249	1.2*	24.5	275	1.4**
ALL OTH.NAT.CAUSES		67.5	879	53.3	691	77.4	1029	1.5**	91.5	1184	1.7**
Inf.&parasit.diseases		5.9	86	4.2	61	8.2	134	1.9**	8.5	118	2.0**
Endocrine dis		6.0	77	4.2	65	8.3	102	2.0**	8.9	94	2.1**
Mental&Neurol Dis.		14.5	184	12.4	148	15.2	196	1.2*	18.3	246	1.5**
Resp.Syst.dis.		16.8	173	13.7	137	18.4	178	1.3**	22.4	238	1.6**
	*Chron.Obs.Pulm.Dis.*	9.2	89	7.1	67	9.7	97	1.4*	13.0	127	1.8**
Digest.Syst.Dis.		12.7	185	10.0	135	13.0	187	1.3*	17.7	274	1.8**
	*Chronic Liver Dis.*	6.0	103	4.0	66	7.1	119	1.8**	9.2	167	2.3**
Genito-urin.Syst.Dis		2.8	26	1.9	17	3.7	46	1.9*	4.2	37	2.2**
Sympt.&Ill-def.		4.6	63	3.2	41	5.4	71	1.7*	7.0	101	2.2**
EXTERNAL CAUSES		23.8	606	21.2	552	25.3	620	1.2*	28.3	702	1.3**
	*Road Accident*	3.9	133	3.5	123	2.2	63	0.6*	5.3	177	1.5**
	*Non transp.acc.(Poison/fall/envir.)*	7.1	130	5.6	98	8.2	172	1.5*	9.6	171	1.7**
	*Suicide*	9.8	266	9.4	265	7.9	208	0.8	11.0	290	1.2*
	*Event of Undet.Intent*	1.4	37	1.1	28	4.7	115	4.2**	1.0	28	0.9
	*Miscell.oth.Ext.causes*	1.5	39	1.5	37	2.3	61	1.5	1.3	36	0.8

*Age-adjusted rates and Potential Years of Life Lost rates per 100.000.*

*ICD chapters with fewer than 200 cases a year are not displayed.

Significance levels of the z test: '**' = p <0.01; '*' = p <0.05; 'ns' = Not significant.

In men, important regional differences are also observed for specific causes: compared to Flanders, Wallonia experiences a 20% excess in cancer death rates (rates ratio = 1.2). Higher death rates are observed in head & neck, lung, liver, pancreas and the miscellaneous other neoplasms, while no regional difference was observed in stomach, colorectal, reproductive organs, haematological and central nervous system cancers. Circulatory diseases present an excess of 30% in Wallonia. The highest excess of death rates (80%) is seen for the miscellaneous group of ‘All other natural causes’, in which the excess ranges between 70 and 110%, depending on the specific causes. The group of external causes presents a 50% excess in Wallonia, with 60% in road as well as in non-transport accidents and 40% for reported suicide.

Compared to Flanders, Brussels presents a 10% excess in male cancer premature death rates (1–74 years). Only liver cancer and “miscellaneous other cancer” show a statistically significant excess with respect to Flanders. Deaths from circulatory diseases and all other natural causes are respectively 20% and 60% higher than in Flanders. Among the external causes, death rates from road accidents in Brussels represent half the Flemish rates, while the rates for non-transport accidents is 50% higher. Reported suicide rates are 20% lower than in Flanders. The death rate for external events of undetermined intent is much higher in Brussels than in the other regions.

In women, excess in Brussels and Walloon’ s rates are observed for the same natural causes of death as for men, with a slightly smaller magnitude, except for head & neck and lung cancer death rates with a 40% excess over Flanders rates in women (against 20% in men). There is no regional difference in the premature breast, gynaecological, colorectal, haematological and CNS cancer death rate. Among external causes, suicide rate in women is 18% higher in Wallonia than in Flanders (versus 40% higher in men).

### Reported and estimated suicide rates

As the categories “event of undetermined intent” (Y10-Y34) and ill-defined and unknown conditions (R96-R99) potentially include suicides, Jougla’s correction [13] has been applied to the reported suicide rates; estimated male suicide rates (1–74 yr) respectively in Flanders, Brussels and Wallonia are 26.1, 24.2 and 36.0 per 100.000 and estimated Potential Years of Life Lost are 763, 607 and 997 per 100.000. Compared to the registered rates (Tables 4 and 5), this represents an increase of 10% in Flanders, 31% in Brussels and 8% in Wallonia.

In women, the estimated suicide rates (1–74 year) after Jougla’s correction are 10.4, 10.6 and 12.3 per 100.000, and the estimated Potential years of Life Lost are 283, 263 and 316 per 100.000, respectively in Flanders, Brussels and Wallonia. Compared to the registered rates (Tables 4 and 5), this represents an increase of 11% in Flanders, 34% in Brussels and 12% in Wallonia.

### Ranking the top 20 causes of deaths

Tables 6 and 7 ranks the causes of deaths according to PYLL in each region, for 2008–2009. It is noteworthy that the ranking would be different based on the indicator used: external causes (suicide, road accident, other accidents) are ranking higher when expressed in PYLL than in rates, while natural causes of deaths (lung cancer, IHD, other circulatory diseases) rank lower. For instance, the suicide rate in men is much lower than the lung cancer rates (respectively 26 and 62 per 100.000), meaning that about 3 times as many men are dying from lung cancer as from suicide. However, in terms of Potential Years of Life lost, suicide ranks first, due to the fact that a great part of the suicides occur in younger people than lung cancer deaths.

**Table 6:** **Top 20 causes of deaths in males by region, Belgium 2008-2009**

*Rank based on PYLL*	*Cause of death Belgium*	*Cause of death Flanders*	*Cause of death Brussels*	*Cause of death Wallonia*
1	Suicide	Suicide	Lung Ca	Suicide
2	Lung Ca	Lung Ca	Isc.Heart Dis.	Lung Ca
3	Road Accident	Road Accident	Suicide	Road Accident
4	Isc.Heart Dis.	Isc.Heart Dis.	Other Circ.Dis.	Isc.Heart Dis.
5	Other Circ.Dis.	Other Circ.Dis.	Digest.Syst.Dis.	Mental&Neurol Dis.
6	Non transp.acc.(Poison/fall/envir.)	Non transp.acc.(Poison/fall/envir.)	Non transp.acc.(Poison/fall/envir.)	Digest.Syst.Dis.
7	Digest.Syst.Dis.	Digest.Syst.Dis.	Resp.Syst.dis.	Other Circ.Dis.
8	Mental&Neurol Dis.	Mental&Neurol Dis.	Mental&Neurol Dis.	Resp.Syst.dis.
9	Resp.Syst.dis.	Resp.Syst.dis.	Event of Undet.Intent	Non transp.acc.(Poison/fall/envir.)
10	Head,neck& oesoph.Ca	Head,neck& oesoph.Ca	Cerebrov.dis.&HTA	Head,neck& oesoph.Ca
11	Cerebrov.dis.&HTA	Cerebrov.dis.&HTA	Head,neck& oesoph.Ca	Sympt&Ill-def.
12	Sympt&Ill-def.	Colorectal Ca	Road Accident	Cerebrov.dis.&HTA
13	Colorectal Ca	Hematol.Ca	Sympt&Ill-def.	Inf.&parasit.diseases
14	Hematol.Ca	CNS Ca	Inf.&parasit.diseases	Colorectal Ca
15	Endocrine dis	Sympt&Ill-def.	Colorectal Ca	Endocrine dis
16	Inf.&parasit.diseases	Endocrine dis	Endocrine dis	Hematol.Ca
17	CNS Ca	Pancreas Ca	Hematol.Ca	Pancreas Ca
18	Pancreas Ca	Inf.&parasit.diseases	Pancreas Ca	CNS Ca
19	Event of Undet.Intent	Event of Undet.Intent	Miscell.oth.Ext.causes	Miscell.oth.Ext.causes
20	Miscell.oth.Ext.causes	Male genital org.Ca	Stomach Ca	Liver Ca

**Table 7:** **Top 20 causes of deaths in females by region, Belgium 2008-2009**

*Rank based on PYLL*	*Cause of death Belgium*	*Cause of death Flanders*	*Cause of death Brussels*	*Cause of death Wallonia*
1	Breast Ca	Breast Ca	Breast Ca	Breast Ca
2	Suicide	Suicide	Lung Ca	Lung Ca
3	Lung Ca	Lung Ca	Other Circ.Dis.	Suicide
4	Other Circ.Dis.	Other Circ.Dis.	Suicide	Other Circ.Dis.
5	Digest.Syst.Dis.	Female genital org.ca	Mental&Neurol Dis.	Digest.Syst.Dis.
6	Mental&Neurol Dis.	Mental&Neurol Dis.	Digest.Syst.Dis.	Mental&Neurol Dis.
7	Resp.Syst.dis.	Resp.Syst.dis.	Female genital org.ca	Resp.Syst.dis.
8	Female genital org.ca	Digest.Syst.Dis.	Resp.Syst.dis.	Cerebrov.dis.&HTA
9	Cerebrov.dis.&HTA	Cerebrov.dis.&HTA	Non transp.acc.(Poison/fall/envir.)	Road Accident
10	Isc.Heart Dis.	Isc.Heart Dis.	Cerebrov.dis.&HTA	Non transp.acc.(Poison/fall/envir.)
11	Road Accident	Road Accident	Isc.Heart Dis.	Isc.Heart Dis.
12	Non transp.acc.(Poison/fall/envir.)	Hematol.Ca	Inf.&parasit.diseases	Chronic Liver Dis.
13	Colorectal Ca	Colorectal Ca	Colorectal Ca	Female genital org.ca
14	Chronic Liver Dis.	Non transp.acc.(Poison/fall/envir.)	Chronic Liver Dis.	Chron.Obs.Pulm.Dis.
15	Hematol.Ca	CNS Ca	Event of Undet.Intent	Inf.&parasit.diseases
16	Chron.Obs.Pulm.Dis.	Chron.Obs.Pulm.Dis.	Endocrine dis	Colorectal Ca
17	Inf.&parasit.diseases	Chronic Liver Dis.	Chron.Obs.Pulm.Dis.	Sympt&Ill-def.
18	Endocrine dis	Endocrine dis	Hematol.Ca	Endocrine dis
19	CNS Ca	Inf.&parasit.diseases	Sympt&Ill-def.	Hematol.Ca
20	Sympt&Ill-def.	Pancreas Ca	Pancreas Ca	CNS Ca

**In men,** at Belgian level, suicide, lung cancer, road accident, ischaemic heart diseases and other circulatory diseases deaths rank highest if expressed in PYLL, while suicide and road accidents rank respectively 5th and 12th if expressed in mortality rates.

The ranking is quite similar for Flanders and Wallonia, but differs in Brussels with respect to external causes: reported suicide ranks 3rd and road accident 12th (expressed in PYLL). However, as events of undetermined intent rank much higher in Brussels, an estimated suicide figure was computed, that ranks 2d in Brussels (instead of 3rd) when expressed in PYLL and 6th (instead of 9th) when expressed in rates.

**In women,** at Belgium level, breast cancer is the leading cause of premature death (expressed in PYLL as well as in rates). It is followed by suicide, lung cancer, other circulatory diseases, diseases of the digestive system. Breast cancer is also the leading cause of death in all 3 regions; it is followed by suicide in Flanders, and by lung cancer in Brussels and Wallonia, where suicide respectively occupies the 4th and 3rd place (ranked on PYLL). After correction, the estimated suicide figure in women ranks 3rd in Brussels (instead of 4th) when expressed in PYLL, and 11th (instead of 15th) if expressed in mortality rates.

### Leading causes by age-group

Tables 8 and 9 details the 5 leading causes of deaths according to age-specific mortality rates within broad age-groups by region.

**Table 8:** **First 5 causes of deaths by age groups and region in males, Belgium 2008–2009**

*Age range*	*Cause of death Belgium*	*Rate*	*Cause of death Flanders*	*Rate*	*Cause of death Brussels*	*Rate*	*Cause of death Wallonia*	*Rate*
1 - 39 yrs	Suicide	16.4	Suicide	16.8	Suicide	10.4	Road Accident	20.8
1 - 39 yrs	Road Accident	15.3	Road Accident	13.9	Non transp.acc.(Poison/fall/envir.)	7.3	Suicide	17.9
1 - 39 yrs	Non transp.acc.(Poison/fall/envir.)	6.8	Non transp.acc.(Poison/fall/envir.)	6.7	Road Accident	6.0	Non transp.acc.(Poison/fall/envir.)	7.0
1 - 39 yrs	Mental&Neurol Dis.	4.1	Mental&Neurol Dis.	2.9	Event of Undet.Intent	5.4	Mental&Neurol Dis.	6.8
1 - 39 yrs	Other Circ.Dis.	2.6	Other Circ.Dis.	2.3	Other Circ.Dis.	2.4	Other Circ.Dis.	3.2
40 - 59 yrs	Lung Ca	53.2	Lung Ca	46.3	Lung Ca	49.8	Lung Ca	67.0
40 - 59 yrs	Isc.Heart Dis.	44.6	Isc.Heart Dis.	35.4	Isc.Heart Dis.	47.9	Isc.Heart Dis.	60.9
40 - 59 yrs	Suicide	40.9	Suicide	34.2	Digest.Syst.Dis.	41.5	Suicide	56.9
40 - 59 yrs	Digest.Syst.Dis.	35.7	Other Circ.Dis.	30.4	Other Circ.Dis.	34.3	Digest.Syst.Dis.	55.5
40 - 59 yrs	Other Circ.Dis.	33.9	Digest.Syst.Dis.	24.2	Suicide	27.5	Mental&Neurol Dis.	40.9
60- 74 yrs	Lung Ca	286.5	Lung Ca	277.4	Lung Ca	255.8	Lung Ca	312.6
60- 74 yrs	Isc.Heart Dis.	210.7	Isc.Heart Dis.	194.5	Isc.Heart Dis.	238.1	Isc.Heart Dis.	237.0
60- 74 yrs	Other Circ.Dis.	184.1	Other Circ.Dis.	177.0	Resp.Syst.dis.	215.7	Resp.Syst.dis.	230.7
60- 74 yrs	Resp.Syst.dis.	178.9	Resp.Syst.dis.	149.1	Other Circ.Dis.	173.7	Other Circ.Dis.	201.2
60- 74 yrs	Cerebrov.dis.&HTA	94.1	Cerebrov.dis.&HTA	84.9	Digest.Syst.Dis.	96.2	Cerebrov.dis.&HTA	114.3

*Age specific rate per 100.000.*

**Table 9:** **First 5 causes of deaths by age groups and region in females, Belgium 2008–2009**

***Age range***	***Cause of death Belgium***	***Rate***	***Cause of death Flanders***	***Rate***	***Cause of death Brussels***	***Rate***	***Cause of death Wallonia***	***Rate***
1 - 39 yrs	Suicide	5.3	Suicide	5.8	Suicide	3.7	Suicide	5.2
1 - 39 yrs	Road Accident	3.6	Road Accident	3.3	Non transp.acc.(Poison/fall/envir.)	2.4	Road Accident	4.8
1 - 39 yrs	Mental&Neurol Dis.	1.8	Breast Ca	1.6	Event of Undet.Intent	2.0	Mental&Neurol Dis.	2.9
1 - 39 yrs	Non transp.acc.(Poison/fall/envir.)	1.7	Hematol.Ca	1.5	Breast Ca	1.7	Non transp.acc.(Poison/fall/envir.)	2.4
1 - 39 yrs	Breast Ca	1.6	Mental&Neurol Dis.	1.2	Mental&Neurol Dis.	1.7	Other Circ.Dis.	1.7
40 - 59 yrs	Breast Ca	35.3	Breast Ca	34.7	Breast Ca	41.3	Breast Ca	34.6
40 - 59 yrs	Lung Ca	25.4	Lung Ca	21.9	Lung Ca	26.1	Lung Ca	31.5
40 - 59 yrs	Digest.Syst.Dis.	17.9	Female genital org.ca	16.0	Other Circ.Dis.	19.3	Digest.Syst.Dis.	28.2
40 - 59 yrs	Other Circ.Dis.	16.5	Suicide	14.1	Female genital org.ca	15.9	Other Circ.Dis.	21.8
40 - 59 yrs	Suicide	15.7	Other Circ.Dis.	13.2	Digest.Syst.Dis.	15.5	Suicide	19.5
60- 74 yrs	Breast Ca	90.8	Breast Ca	94.0	Lung Ca	97.0	Other Circ.Dis.	106.8
60- 74 yrs	Other Circ.Dis.	90.5	Other Circ.Dis.	81.9	Breast Ca	90.0	Resp.Syst.dis.	101.7
60- 74 yrs	Resp.Syst.dis.	79.3	Isc.Heart Dis.	68.5	Other Circ.Dis.	90.0	Lung Ca	93.6
60- 74 yrs	Lung Ca	78.2	Lung Ca	67.6	Resp.Syst.dis.	87.7	Breast Ca	85.1
60- 74 yrs	Isc.Heart Dis.	72.8	Resp.Syst.dis.	66.4	Isc.Heart Dis.	81.6	Isc.Heart Dis.	78.7

*Age specific rate per 100.000.*

Under the age of 40, suicide and accidents rank first in all regions and in both sexes.

Lung cancers and ischemic heart diseases are the main causes in men after 40, while suicide progressively occupies a lower place.

Between 40 and 59 years, breast and lung cancer deaths occupy the first places in women in all regions. In women aged 60–74, the ranking and the specific rates differ considerably between regions: breast cancer ranks first only in Flanders (in term of mortality rates), with slightly higher rates than in the other regions. The other circulatory and respiratory diseases rank first in Wallonia, with much higher mortality rates than in Flanders. Lung cancer deaths mortality rates are considerably higher in Brussels (where it ranks first) and Wallonia than in Flanders.

### Evolution over time

Tables 10 and 11 shows the evolution of the overall and cause specific age-standardised death rates (1–74 years) and PYLL across 4 time periods (separated by 5 years): 1993–1994, 1998–1999, 2003–2004 and 2008–2009. For the period 1998–1999, only overall and cancer death rates are displayed because of a lack of reliability for the other causes of death.

**Table 10:** **Evolution of premature deaths in males by main causes in Belgium, across 4 time-period, 1993–2009**

*Cause of death*	*RATES 1993-1994*	*RATES 1998-1999* ^*$*^	*RATES 2003-2004*	*RATES 2008-2009*	*15 years change RATES*	*PYLL 1993-1994*	*PYLL 1998-1999*	*PYLL 2003-2004*	*PYLL 2008-2009*	*15 years change PYLL*	*Sign level change in rates* ^*$$*^
ALL PREMATURE	661.9	601.6	522.0	466.3	**-29.6%**	8834	8020	7109	6410	**-27.4%**	**
NATURAL CAUSES	584.2	.	456.9	404.7	**-30.7%**	6375	.	5111	4615	**-27.6%**	**
ALL NEOPLASMS	238.6	216.7	190.3	175.1	**-26.6%**	2610	2343	2097	1914	**-26.7%**	**
Head,Neck & oesoph.Ca	21.0	19.9	18.3	16.6	**-20.8%**	317	288	254	219	**-30.8%**	**
Stomach Ca	9.8	7.8	6.3	5.1	**-48.4%**	97	81	72	56	**-42.4%**	**
Colorectal Ca	20.4	19.4	16.4	15.6	**-23.6%**	197	188	154	152	**-22.8%**	**
Liver Ca	5.1	5.1	5.4	5.9	**16.0%**	54	53	58	64	**18.9%**	*
Pancreas Ca	9.3	9.3	8.7	9.0	**-3.0%**	101	93	94	92	**-9.0%**	ns
Lung Ca	94.5	81.1	67.5	61.9	**-34.6%**	944	803	682	630	**-33.3%**	**
Male genital org.Ca	14.7	13.0	10.0	8.8	**-40.4%**	97	86	67	65	**-33.3%**	**
CNS Ca	7.4	6.7	5.5	5.9	**-20.3%**	133	115	105	106	**-20.3%**	**
Hematol.Ca	15.6	13.9	12.7	11.5	**-25.9%**	214	180	154	138	**-35.4%**	**
Other Neoplasms	40.8	40.7	39.5	34.9	**-14.5%**	456	457	458	391	**-14.3%**	**
CIRCULATORY DISEASES	193.7	.	136.4	109.6	**-43.4%**	1893	.	1400	1176	**-37.9%**	**
ALL OTH.NAT.CAUSES	151.9	.	130.3	120.0	**-21.0%**	1871	.	1614	1525	**-18.5%**	**
Chron.Obs.Pulm.Dis.	38.1	.	25.0	21.5	**-43.6%**	288	.	188	170	**-41.2%**	**
Chronic Liver Dis.	14.8	.	13.0	13.5	**-8.9%**	267	.	224	217	**-18.7%**	*
Remaining Other Natural Causes	89.1	.	81.2	76.3	**-14.4%**	1119	.	1056	1022	**-8.7%**	**
EXTERNAL CAUSES	77.7	.	65.0	61.5	**-20.8%**	2459	.	1997	1795	**-27.0%**	**
Road Accident	25.7	.	17.5	14.4	**-44.0%**	966	.	664	527	**-45.5%**	**
Non transp.acc.(Poison/fall/envir.)	14.4	.	14.8	15.8	**9.7%**	396	.	350	358	**-9.6%**	*
Suicide	29.5	.	28.1	26.2	**-11.4%**	871	.	839	760	**-12.7%**	**
Event of Undet.Intent	3.1	.	2.0	2.9	**-3.6%**	95	.	65	85	**-11.0%**	ns
Miscell.oth.Ext.causes	5.0	.	2.4	2.3	**-54.6%**	131	.	76	65	**-50.2%**	**

*Age-adjusted rates and age-adjusted PYLL rates per 100.000.*

*15 years changes in rates and PYLL are displayed in bold*.

*$.1998-1999 figures by detailed COD not shown for lack of reliability (except for neoplasms).*

$$.Significance level of the change in Rates: ns 'Not Significant' * ' < 0.05' ** ' < 0.01'.

**Table 11:** **Evolution of premature deaths in females by main causes in Belgium, across 4 time-period, 1993–2009**

*Cause of death*	*RATES 1993-1994*	*RATES 1998-1999* ^*$*^	*RATES 2003-2004*	*RATES 2008-2009*	*15 years change RATES*	*PYLL 1993-1994*	*PYLL 1998-1999*	*PYLL 2003-2004*	*PYLL 2008-2009*	*15 years change PYLL*	*Sign level change in rates* ^*$$*^
ALL PREMATURE	322.2	296.9	267.2	251.2	**-22.0%**	4358	4064	3630	3440	**-21.1%**	**
NATURAL CAUSES	292.1	.	243.0	227.4	**-22.2%**	3525	.	2988	2834	**-19.6%**	**
ALL NEOPLASMS	128.5	121.3	111.3	111.0	**-13.7%**	1749	1626	1468	1445	**-17.4%**	**
Head,Neck & oesoph.Ca	3.5	3.7	3.9	3.6	**3.0%**	53	53	55	46	**-13.2%**	ns
Stomach Ca	3.6	3.1	2.3	2.2	**-39.2%**	38	36	27	30	**-21.6%**	**
Colorectal Ca	13.5	12.0	10.2	9.8	**-27.3%**	146	131	106	107	**-27.1%**	**
Liver Ca	2.1	2.5	2.2	2.4	**16.2%**	25	24	24	23	**-5.8%**	ns
Pancreas Ca	5.7	6.3	5.4	5.5	**-2.0%**	57	63	50	57	**0.6%**	ns
Lung Ca	13.1	13.9	15.9	19.7	**50.2%**	175	183	210	248	**41.9%**	**
Breast Ca	33.5	31.6	26.9	25.2	**-24.6%**	517	482	411	364	**-29.5%**	**
Female genital org.ca	17.3	14.9	13.7	13.4	**-22.8%**	232	199	172	171	**-26.3%**	**
CNS Ca	5.6	4.7	4.0	4.0	**-29.0%**	103	84	70	76	**-26.1%**	**
Hematol.Ca	9.8	8.6	8.0	7.4	**-24.9%**	144	116	105	99	**-31.0%**	**
Other Neoplasms	20.9	19.9	18.8	17.7	**-15.0%**	260	254	237	223	**-14.2%**	**
CIRCULATORY DISEASES	88.8	.	61.7	49.0	**-44.9%**	799	.	617	510	**-36.2%**	**
ALL OTH.NAT.CAUSES	74.8	.	70.0	67.5	**-9.8%**	977	.	902	879	**-10.0%**	**
Chron.Obs.Pulm.Dis.	10.2	.	9.3	9.2	**-9.8%**	101	.	92	89	**-11.7%**	*
Chronic Liver Dis.	7.7	.	6.0	6.0	**-22.3%**	134	.	107	103	**-23.0%**	**
Remaining Other Natural Causes	52.3	.	47.5	46.4	**-11.2%**	657	.	601	601	**-8.5%**	**
EXTERNAL CAUSES	30.1	.	24.2	23.8	**-20.9%**	832	.	642	606	**-27.2%**	**
Road Accident	8.2	.	5.2	3.9	**-51.9%**	282	.	174	133	**-52.6%**	**
Non transp.acc.(Poison/fall/envir.)	6.8	.	6.5	7.1	**5.1%**	152	.	118	130	**-14.5%**	ns
Suicide	10.7	.	10.1	9.8	**-9.2%**	287	.	280	266	**-7.5%**	*
Event of Undet.Intent	1.4	.	0.7	1.4	**0.6%**	38	.	20	37	**-3.3%**	ns
Miscell.oth.Ext.causes	2.9	.	1.7	1.5	**-47.4%**	73	.	51	39	**-45.7%**	**

*Age-adjusted rates and age-adjusted PYLL rates per 100.000.*

*15 years changes in rates and PYLL are displayed in bold*.

*$.1998-1999 figures by detailed COD not shown for lack of reliability (except for neoplasms).*

$$.Significance level of the change in Rates: ns 'Not Significant' * ' < 0.05' ** ' < 0.01'.

Overall premature death rates **in men** steadily decreased over the 4 time periods (Tables 10 and 11, 5th column), reaching a 29.6% decrease between the first (1993–4) and the 4th period (2008–2009). Mortality rates resulting of natural causes decreased faster (-30.7%) than for external causes (-20.8%). Within the natural causes, circulatory diseases death rates decreased the fastest (-43.4%), followed by the neoplasms (-26.6%) and the other natural causes (-21.0%). The decrease in specific cancer death rates greatly differs by organ: change was the highest for stomach (-48.4%), followed by genital organs (-40.4%) and lung (-34.6%) cancers. Colorectal, head & neck and CNS cancers decrease by more than 20%. Pancreas cancer death rates remain stable. Finally, an increase was observed for liver cancer (+16%).

Within the external causes, road accident decreased by 44%; suicide rate slightly decreased (-11%), while the non-transport accident increased (by 10%).

Changes over time are nearly of the same magnitude when expressed in PYLL or in mortality rates. However, for some conditions, the decrease in PYLL was faster than the decrease in rates, meaning that the mortality reduction concerned more younger than older people: this was the case for head & neck and haematological cancers, non-transport accident and event of undetermined intent.

**In women** also, the overall premature death rates has steadily declined over the 4 time periods, reaching a 22% decrease between the 1st and the 4th period. Rates resulting of natural or external causes declined similarly.

Within the natural causes, circulatory diseases deaths rates decreased much faster (-44.9%) than those due to neoplasms (-13.7%) or to the other natural causes (-9.8%). Among deaths resulting from cancer, the decrease was the fastest for stomach (-39.2%). Colorectal, breast, haematological, gynaecological and CNS decreased by 20-30%. Pancreas cancer death rates remain stable. The most worrying feature is the 50.2% increase in the lung cancer death rate. As in men, liver cancer increased by 16%.

Within the external causes, road accident decreased by 51.9%; the slight changes in suicide rate (-9%) or in non-transport accident (by 5%) are not significant.

PYLL decreased faster than mortality rates from breast, gynaecological, haematological, liver and lung cancers, and non-transport accident deaths, suggesting a more favourable evolution in young women. The reverse was seen for stomach cancer deaths.

## Discussion

Recently published data on causes of death were used to analyse the burden, the ranking, the regional disparities and the 15 years evolution of premature mortality in Belgium, filling in a 12 years gap in Belgian figures. The addition of the PYLL indicator to the classical age-adjusted mortality rates completes the picture by weighting the burden of each condition with the loss of quantity of life. Ranking the causes of premature death based on PYLL helps setting up priorities for policy-makers. However, since ranks are ordinal variables, they provide no quantitative information over the burden of the causes; therefore, one should always consider the value of the PYLL (or of the rates) for a specific cause, above its ranking when setting up priorities or to make regional comparison.

There are well-known shortcomings with mortality data: they do not capture morbidity nor quality of life aspects, they rely on death certificate data of which quality is questionable for certain age groups and certain causes, they are coded using a classification which evolves over time. However the advantages of vital statistics are evident: they are routinely available, exhaustive, and they provide information to support health policy. They are of particular interest for planning and evaluating interventions aiming to reduce premature mortality.

### Quality of death registration data

Aelvoet and all [20, 21] investigated the quality of the coding of death certificates data in Belgium until 1997. The conclusion was that the quality had appreciably improved since 1993, when the responsibility of the coding became more centralized and was assigned to the regional Health Agencies (currently two: one is coding for Flanders and Brussels, the second is coding for Wallonia). Caution was however recommended for analysing long-time trends and studying older age groups. By restricting the present analysis to data from 1993 to 2009 and by limiting the scope to premature deaths (1–74 years), the more questionable time periods and age groups were avoided.

Despite coordination meetings between the regional and national levels aiming to minimize the variability in regional practices, the coding of death certificates in 2 regional Health Agencies can still result in differences in the implementation of ICD rules. This issue will be discussed along with the interpretation of regional disparities. In 1998 a new model of death certificate was introduced in Belgium: it is conform to the WHO model and is suitable to be filled in by the certifying physician. Hence, less coding errors are to be expected after the running-in period.

The certification of the cause of death by the physician represents a cornerstone in the production of mortality statistics, it is thus important that it achieves a sufficient quality level. It was not the purpose of the present study to focus on the quality of the certification, so the quality evaluation was limited to the calculation of the percentage of imprecise codes, as defined by the WHO [22]. This latter is still representing 12% in 2008–2009 (8% for the age groups <75 years). As a consequence, efforts should still be made to improve the quality of the deaths certificates by insuring continuous training in death certification for the medical doctors and large dissemination of certification guidelines

Moreover, regional differences in certification might induce differences in observed rates, For instance, the statement that the category “events of undefined intend” represents a higher rates in Brussels than in Flanders may be considered to be at least partly due to regional difference in the certification rather than in coding procedures, since the death certificates of Flanders and Brussels are coded by the same Regional Authority.

The present study covers 2 versions of the ICD [9, 10], ICD-10 being implemented in 1998 in Belgium. Janssen and all [23] examined the effect of coding changes within ICD revisions. They evaluated the proportion of discontinuity for the ICD_9 to ICD_10 change to be lower than the former changes, namely 4.6%, and affecting more specifically dementia, other circulatory diseases, symptoms and ill-defined conditions, other diseases (than those specifically studied), and falls. Specific cancers deaths were hardly affected by those changes.

After evaluation of the data used in the present study, it was decided not to publish the 1998–9 cause specific results, except for neoplasms, because of instability in coding practices. We noticed a substantial improvement after year 2000, partly due to the use of automated coding programs. During the analysis inconsistencies were detected in time series and in regional patterns of mental and neurological diseases (data not shown). This is due to the fact that the codes used for dementia (mainly F01, F03 and G30), are dispersed across 2 different chapters of the ICD. The choice of a specific dementia code relies both on the diagnosis reported (by the physician who completes the death certificate) and on the coding rules of the Regional Agency, and has some impact on the death rate for each of those disease groups. The specific study of dementia pattern was out of the scope of this paper, so that it was decided to pool the chapters about mental and neurological diseases together.

### The PYLL indicator

The ranking of the conditions depends on the choice of the indicator. The PYLL indicator emphasizes causes of death occurring at younger ages (mainly external causes) and is generally considered more appropriate to study the burden of premature death and to help setting up priorities. Depending on the focus of the study, various age limits have been used to calculate the PYLL indicator [1–5, 24], leading to different rankings of the causes of death. For instance studies focusing on productivity losses only include productive ages, mainly 20–65 years, while in studies of premature deaths the upper cut-off ranges from 65 to the age corresponding to life expectancy, with inclusion or not of infant deaths.

In the current study the upper cut-off was set at 75 years for two reasons: reported conditions for deaths occurring after 75 years are generally less reliable because of more frequent competing causes of death in older people; moreover the choice of a 75 year upper- limit is consistent with the recent definition of avoidable mortality [25].

The inclusion of infant deaths is also a matter of debate. They have been excluded from the present study, as recommended by Romeder [5], because infant mortality is due to causes that are very specific to this age group. Moreover, the weight given to infant deaths account for almost 75 years in the computed PYLL, which is probably an overestimation of the societal weight of those deaths in comparison of a death occurring in young adulthood. Finally, more appropriate indicators exist that specifically address death in this particular age group.

### Interpretation of findings

Compared to the other EU15 countries, overall premature deaths rates and PYLL are quite high in Belgium. This comparison with similar countries highlights potential gains in life expectancy.

The male to female premature mortality ratio [2] is much higher than the one observed in all age mortality (1.6), suggesting that premature mortality contributes more to the gender difference in life expectancy than mortality at older ages.

Among men, the leading causes of premature deaths ranked according to PYLL are suicide, lung cancer, road accident, ischemic heart disease and other circulatory disease. Among women, breast cancer still occupies the top position, followed by suicide, lung cancer, other circulatory diseases, and digestive diseases (more than half of this latter being due to chronic liver disease). It is noteworthy that almost all of those causes are also considered as “avoidable death”, meaning sensitive either to the health system or to public health interventions. There is room for improvement by further reducing those avoidable deaths.

### Regional differences

The ranking of causes of deaths is roughly the same for Flanders and Wallonia, but differs somehow in Brussels, where road accidents rank much lower than in the other regions; suicide in men also occupies a lower place than in the other regions. Regional disparities in overall and cause specific mortality for all ages [6, 9, 26, 27], in premature mortality [8] and in avoidable mortality [28, 29] have been documented in the past (until 1997). Since World War II, Wallonia always experienced a clear disadvantage in male mortality, mostly due to deaths related to circulatory diseases, neoplasms, respiratory diseases, chronic liver diseases, external causes and infectious diseases. At the contrary to our finding, in the older studies, stomach cancers were higher in Flanders than in the two other regions [7] and most of the other natural causes didn’t show significant differences.

The present work updates, at the hand of the newly available vital statistics, the information about the regional disparities in overall and cause specific premature mortality. An important Walloon excess in male overall premature mortality as compared to the Flemish rates (Rate ratio: 1.4) is still observed, for the same specific causes as in previous studies. It is noteworthy that the Wallonia-to-Flanders rate ratio for premature death is much higher than the rate ratio for all ages mortality, which is only 1.2 in 2009 (source = spma https://s9xjb.wiv-isp.be/SASStoredProcess/guest?_program=/SPMA/SP/allcaus). Again, those large regional disparities highlight the possible room for improvement. Such regional disparities could be explained by differences in the socio-economic context, in health policies and to some extent in health care practices, by environmental factors and cultural differences in health behaviour. The socio-economic context is worse in Wallonia than in Flanders; however, an analysis of the other factors and particularly an evaluation of the health policies could highlight some causes of the observed differencesthat could be vulnerable to policy interventions.

The present study also yielded some new findings, e.g. the former regional difference in stomach cancer mortality (with Flanders at a disadvantage) has totally disappeared, probably because of a change in the way of conserving food. Beside this, a 80-100% excess was observed in Wallonia as compared to Flanders for many natural causes (grouped into ICD chapter, namely mental and neurological, digestive, endocrine diseases). This had never been described before: at the contrary, Van Houte-Minet [6] explicitly mentioned that no significant regional difference was observed for those specific causes during the period 1970–72. Other previous studies did not specifically examine regional disparities for those causes.

Men in Brussels experience a premature mortality rate intermediate between the Flemish and Walloon rates, with a main Brussels-to-Flanders excess in the group of “other natural causes” (+60%), then in the circulatory diseases (+20%), and cancers (10%). While no Brussels-to-Flanders difference is observed in the overall rate of external causes, large differences are observed in the specific external causes of deaths: for evident reasons, the road accidents rate is much lower than in the other regions. The suicide rate, even after the Jougla’s correction, is lower in Brussels than in the other regions and no clear explanation for this could be found. It is noteworthy that the category of non-transport accident (in our classification: fall, poisoning and environmental accidents) is higher than in the other regions, which should be further explored: there are possibly more infrastructure problems in housing in big towns, however suicide declared as accidental poisoning cannot be excluded. The unexplained high rate of events of undetermined intent in Brussels requires caution in the interpretation of specified external causes.

**Among women,** the overall Wallonia to Flanders rate ratio for premature death is slightly less pronounced than in men (RR = 1.3). The RR for cancer is only 1.1 (10% excess); this is mainly attributable to smoking related cancers, namely head & neck and lung cancers in which the Walloon excess reaches 40%. Rate ratios in other causes show the same pattern as in men.

As it was the case for men, premature mortality rates for women in Brussels are in between those of Flanders and Wallonia, both for overall mortality and for natural cause mortality. For external causes, a similar pattern is observed as in men, with a lower rate of road accidents and suicide, and a higher rate of non-transport accident.

Although coding was processed in two separate health agencies, it is unlikely that this could explain much of the observed regional differences. The difference in overall mortality is anyway not related to different coding rules. Second, as the category “symptoms and ill-defined causes” represents a higher proportion of deaths in Wallonia (2.6% in 2008–9) than in Flanders (1.7%) and is almost the same as in Brussels (2.5%), any reclassification of poorly into better-defined chapters would result in an increase of specific death rates, that would be larger in Wallonia than in Flanders, still accentuating the regional differences. Finally, differences at highly aggregated levels, such as head of ICD chapters, could hardly be due to variations in coding practices. This was only the case for mental and neurological diseases, a problem that was solved by pooling the categories.

Only a few of single causes have been studied in detail, namely the cancers, circulatory diseases, COPD, chronic liver diseases and external causes. The coding of cancer type is unlikely to be subject to regional differences; regional differences in COPD and chronic liver diseases deaths have since long been documented and explained by life styles and working environment differences. However, a regional coding bias is not excluded for specific circulatory diseases, so that it is preferable to only compare the pooled category of circulatory diseases.

The category external causes is also prone to create differences in coding and should be examined with caution. A clear regional bias is observed for the category “event of undetermined intent”, which is much higher in Brussels. Technics of correcting for not-at-random missing values could be used to further explore the apparent differences in external deaths.

Measuring changes in the magnitude of the regional disparities over time was out of the scope of this study, and methodological differences between previous studies and this one ask for more in depth research.

### Evolution of the national rates over time

Among men, a substantial decrease is observed in death rates due to road accidents, probably as a result of drastic measures in the road security policy. Regarding death related to tobacco use, a major decrease is observed in circulatory diseases, larger than the one observed for head & neck and lung cancer. This discrepancy may suggest the influence of additional factors as changes in diet, prevention and medical treatment of hypertension. Despite the progress observed in the last decade, road accidents, lung cancers and circulatory diseases remain the main causes of potential years of life lost, and policy-makers should pursue efforts to further reduce them.

The important decrease in stomach cancer likely results from a change in the methods of food preservation, leading to a reduction in salt consumption. The reduction in haematological and reproductive organ cancer death rates is possibly due to improvement of chemotherapy schemes.

Suicide has hardly declined and is nowadays the main cause of premature deaths in men at national level, when measured in PYLL; moreover, if the correction for underreporting is applied, suicide far exceeds all other single causes of death in men, except in Brussels.

The slight increase of liver cancer mortality should be further explored; this could result from a change in reporting, but increased viral hepatitis or alcohol consumption could also play a role.

The stability in non-transport accidents (fall, poisoning, environment) deaths should be checked, as it has been reported that the change in ICD version impact the way of coding for those specific causes [23].

Among women, the 15 years decrease in overall mortality is slightly lower than in men (22% decrease in mortality rates). Already lower mortality rates are probably harder to improve. However, attention should be paid to the fact that the decrease in cancer rates (-14%) is much slower than in circulatory diseases (-45%), and slower than in men (-27%). The 50% increase in lung cancer rates and the stability in head & neck cancer rates are responsible for this slower evolution. The smoking behaviour of the women could potentially explain this evolution. As in men, the discrepancy between the evolution in lung cancer and in circulatory diseases mortality suggest that smoking is not the only factor influencing the latter rate.

## Conclusion

An impressive improvement in premature mortality over the last 15 years has contributed to an increase in life expectancy. Our study shows that mortality declined for most causes, but the 50% increase in female lung cancer mortality is an alarming evolution. The comparison with EU15 countries proofs that there is room for improvement. This is even more the case for the Walloon region with distinct higher overall and cause specific mortality. Even if the less favourable socio-economic context in Wallonia certainly contributes to these differences, an in-depth evaluation of health policies and health system performance would give some insights to the roots of the regional disparities and contribute to improve the situation.

## Authors’ original submitted files for images

Below are the links to the authors’ original submitted files for images. Authors’ original file for figure 1

## Abbreviations

PYLL: Potential years of life lost
WHO: World Health Organisation
Statistics Belgium: Directorate-general statistics Belgium
ICD: International classification of diseases
CNS Ca: ‘Central nervous system cancer’
CPOD: Chronic pulmonary obstructive disease
Isc.Heart Dis.: ‘Ischemic heart diseases’
Cerebrov.dis.&HTA: ‘Cerebrovascular disease and hypertension’
Sympt&Ill-def: Symptoms and ill-defined conditions
Non transp.acc.(Poison/fall/envir.): ‘Non transport accidents (Poisoning, fall, environmental accidents).
