# Supplementary material for: Premature mortality in Belgium in 1993-2009: leading causes, regional disparities and 15 years change
Source: Arch Public Health. 2014 Oct 1;72:34. doi: 10.1186/2049-3258-72-34 (PMC4200135; doi:10.1186/2049-3258-72-34)
Supplement: Supplementary file 1 — Authors’ original file for figure 1 [file 13690_2014_5050_MOESM1_ESM.docx]

Figure 1
